# Screening for personality disorder in incarcerated adolescent boys: preliminary validation of an adolescent version of the standardised assessment of personality – abbreviated scale (SAPAS-AV)

**DOI:** 10.1186/1471-244X-12-94

**Published:** 2012-07-30

**Authors:** Mickey Kongerslev, Paul Moran, Sune Bo, Erik Simonsen

**Affiliations:** 1 Department of Child and Adolescent Psychiatry, Region Zealand, Roskilde, Denmark; 2 Psychiatric Research Unit, Region Zealand, Toftebakken 9, 4000, Roskilde, Denmark; 3 King’s College London, Health Services & Population Research Department, Institute of Psychiatry, London, United Kingdom

**Keywords:** Personality disorder, Personality assessment, Screening, Psychiatric epidemiology, Adolescence, Aggression, Juvenile offenders

## Abstract

**Background:**

Personality disorder (PD) is associated with significant functional impairment and an elevated risk of violent and suicidal behaviour. The prevalence of PD in populations of young offenders is likely to be high. However, because the assessment of PD is time-consuming, it is not routinely assessed in this population. A brief screen for the identification of young people who might warrant further detailed assessment of PD could be particularly valuable for clinicians and researchers working in juvenile justice settings.

**Method:**

We adapted a rapid screen for the identification of PD in adults (Standardised Assessment of Personality – Abbreviated Scale; SAPAS) for use with adolescents and then carried out a study of the reliability and validity of the adapted instrument in a sample of 80 adolescent boys in secure institutions. Participants were administered the screen and shortly after an established diagnostic interview for DSM-IV PDs. Nine days later the screen was readministered.

**Results:**

A score of 3 or more on the screening interview correctly identified the presence of DSM-IV PD in 86% of participants, yielding a sensitivity and specificity of 0.87 and 0.86 respectively. Internal consistency was modest but comparable to the original instrument. 9-days test-retest reliability for the total score was excellent. Convergent validity correlations with the total number of PD criteria were large.

**Conclusion:**

This study provides preliminary evidence of the validity, reliability, and usefulness of the screen in secure institutions for adolescent male offenders. It can be used in juvenile offender institutions with limited resources, as a brief, acceptable, staff-administered routine screen to identify individuals in need of further assessment of PD or by researchers conducting epidemiological surveys.

## Background

Converging research has demonstrated that there is a disproportionately high prevalence of mental disorders in young offender samples [1,2], when compared with community youth [3] or adult prison populations [4]. Current estimates suggest that up to 75% of incarcerated juvenile offenders meet criteria for one or more mental disorders [5]. Such research findings, combined with reports of a high prevalence of undiagnosed and untreated physical disorders [6,7], high rates of suicide and suicide attempts [8], and elevated mortality rates associated with early death due to unnatural causes [9], have led the American Academy for Child and Adolescent Psychiatry [5], as well as other experts [10-13] to call for the development and implementation of standardised and validated routine screening instruments in juvenile correctional facilities, lest the mental healthcare needs of young offenders are overlooked and untreated.

Personality disorders (PDs) are amongst the most common mental disorders in populations of incarcerated adults [4], and yet they remain understudied disorders in juvenile offenders. Few psychiatric morbidity surveys have included assessment of PDs, and in those studies that have, prevalence estimates reported for any PD have ranged from 36% [14] to 88% [15]. The majority of research on personality pathology in juvenile offenders has focused on the constructs of ‘psychopathy’ [16] or ‘callous-unemotional traits’ [17], which are psychological constructs with important implications for risk assessment, but not currently included in the ICD-10 [18] and DSM-IV-TR [19]. Concerns about the stigma associated with a PD diagnosis, coupled with doubts about the stability of personality traits in adolescence have led to a reluctance to diagnose PD in young people. Furthermore, both the ICD-10 and DSM-IV-TR, although allowing for diagnosing PDs in young people below the age of 18 years, somewhat discourage this practice, by explicitly stating that it is unlikely or only in relatively unusual circumstances that this will be appropriate. Yet, as pointed out by Westen and Chang [20] such statements were based on a paucity of data on PDs in adolescence. Indeed, within the last decade, accumulating evidence suggests that PDs can be diagnosed just as reliably and with as much validity as in adults [21-25]. These findings may also be reflected in the forthcoming revisions of ICD and DSM, where the proposals for new diagnostic criteria, based on a dimensional approach to PDs, are likely to remove references to age criteria [26,27]. Furthermore, research findings in clinical and community samples of young people show that the presence of PD worsens long-term prognosis of co-morbid Axis I disorders [28], and is associated with impulsive violence and completed suicide [29], substance abuse [30], elevated risk of development of major mental disorders, suicidality in adulthood [31,32], and significant psychosocial impairments and distress [33,34]. In offender populations, PD in adolescence is associated with elevated risk of premature and unnatural death [9], and, in adult prison populations, criminal recidivism and violence [35,36]. Taken together, these data suggest that there is much to be gained from the early detection of PD among young offenders and the provision of targeted early intervention programmes [37,38], at a time when personality is presumably more malleable [33,39].

Assessment of PD in juvenile offenders poses a considerable challenge to clinicians and researchers. The use of semi-structured diagnostic interviews is time-consuming, and often requires specialised training. A brief, simple, and acceptable screening instrument, which could be rapidly administered by non-specialists to identify adolescents in need of further assessment for PD, could overcome some of these obstacles. The American Academy of Child and Adolescent Psychiatry’s practice guidelines recommends self-report instruments for screening purposes in juvenile justice settings [7]. A number of self-report screens for PDs are available [40], but few of them have been validated in adolescent offender samples, or only target one specific type of PD [41]. We suggest that a mini-interview covering the broad domain of PD is preferable because self-report questionnaires require ability to concentrate and read written questions, which can be difficult for many juvenile offenders [2,10]. The necessity for breadth of coverage is driven by the fact that many different types of PDs are prevalent in adolescent samples [42].

To our knowledge, there are currently no interview-based PD screens available for use with adolescents. Several interview-administered screens for PDs have been developed for use with adults [40,43]. The Standardised Assessment of Personality – Abbreviated Scale (SAPAS) [44] is the shortest and simplest to use. It consists of eight yes/no questions, requires no administration training for clinicians, and only simple guidance for lay interviewers. The SAPAS was developed from the semi-structured interview Standardised Assessment of Personality [45,46], and validated in a sample of psychiatric patients, where it was found to have good psychometric properties, correctly identifying the presence of personality disorder in 90% of the patients, with a sensitivity and specificity of 0.94 and 0.85 respectively [44]. Subsequently the SAPAS has been validated in samples consisting of patients with substance abuse [47,48], depression [49,50], and probationers [51], confirming the findings from the original validation study. Based on this, we hypothesised that with minor adaptations the SAPAS might be a useful screen for PDs in juvenile justice settings.

To this end, we designed and conducted a prospective study to investigate the psychometric properties and diagnostic accuracy of an adolescent version of the SAPAS (SAPAS-AV) in a sample of incarcerated adolescent boys. The primary aim was to examine the discriminatory performance of SAPAS-AV against a categorical criterion diagnosis of any PD on the Structured Clinical Interview for DSM-IV Axis II Personality Disorders (SCID-II) [52], including estimating sensitivity and specificity for different cut-scores. We also assessed reliability in terms of internal consistency and test-retest reliability for a period of 9 days. Because future revisions of the classification schemes are likely to include dimensional representations of PD, we furthermore examined the convergent validity of the SAPAS-AV against dimensional PD scores on the SCID-II.

## Method

### Participants

Participants were recruited from 3 secure institutions and 1 prison ward for juvenile offenders in Denmark. Staff approached eligible participants when the principal investigator (MK) was visiting the sites. Those interested in participating received both written and verbal information about study aims and procedures from the first author (MK) before they finally decided on whether they whished to participate or not, and written informed consent was obtained from all the young people participating. Two secure institutions were chosen as primary recruitment sites due to their close geographical location. The principal investigator circulated between these two sites, during the recruitment period. The other two sites were visited only once. Eligible participants were remanded (pre- and post-trial) or sentenced boys, aged 15–18 years inclusive, sufficiently fluent in Danish, and willing and able to give informed consent. Exclusion criteria were: (1) profound mental retardation; (2) alcohol and substance intoxication or withdrawal symptoms on days of assessment; and (3) active psychosis on days of assessment.

### Measures

#### Screen

The Standardised Assessment of Personality–Abbreviated Scale: Adolescent Version (SAPAS–AV; see Appendix 1) was adapted from the Danish version of the SAPAS [44,47]. Initial refinement of the language used in the SAPAS-AV took place after piloting the SAPAS on 10 adolescent boys and girls from a secure institution and a psychiatric outpatient clinic. The purpose of this pilot was to examine whether the respondents understood the questions and found the interview acceptable. On the basis of this pilot, 2 items were slightly modified to ease comprehension. Item 5 of the original SAPAS was changed from *Are you normally an impulsive sort of person* to *Are you normally an impulsive sort of person, who generally acts before thinking*. This modification was made to enhance comprehension, because most young people in the first pilot were unsure about the actual meaning of the word ‘impulsive’. Item 7 of the SAPAS was reworded from *In general*, *do you depend on others a lot* to *In general, and compared to your peers, do you depend on others a lot* in the SAPAS-AV. The rationale for the rewording of this item was that although adolescence is developmentally a phase of beginning autonomy, most adolescents still depend a lot on parents and other adults, which is expectable and normative. Accordingly it became necessary to highlight in this question that it was in comparison to their peers, to make it more likely that the question would target potential personality problems rather than developmentally normative personality traits and behaviours. The acceptability of the refined version was then tested on a different sample comprised of 5 adolescent boys from a secure institution and 5 adolescent girls from a psychiatric outpatient clinic. On the basis of this testing, comprehension was judged to be acceptable.

Like its adult counterpart, the SAPAS-AV is a mini-interview consisting of eight dichotomously rated items, corresponding to descriptive statements about a person, essentially covering the broad multidimensional domain of DSM-IV PDs. The items are worded as questions to be answered either *yes* or *no*. No interpretation is placed on the response on behalf of the interviewer. Responses are summed to give a total score ranging from 0 to 8. The screen can usually be completed in less than five minutes.

#### Reference standard

The Structured Clinical Interview for DSM-IV Axis II Personality Disorders (SCID-II) [52] was chosen as reference standard because it is internationally well known and widely used in research with both adults [53] and adolescents [41]. The SCID-II is a semi-structured interview designed to assess the DSM-IV PDs, including two PDs in Appendix B of DSM-IV-TR, and PD not otherwise specified. As in previous studies on the SAPAS [44,51] we only considered the 10 main DSM-IV-TR PD categories in this report. The interview consists of 119 sets of questions plus additional questions to assess antisocial PD. The questions correspond to the specific diagnostic criteria for each personality disorder. Each question is rated as: 1 = absent/false; 2 = subthreshold; 3 = threshold/true; or ? = inadequate information. The number of questions rated positive (rated 3) for each specific PD is summed to yield a dimensional score. If the dimensional score equals or exceeds the DSM-IV-TR diagnostic threshold for each PD a categorical diagnosis can be made. The interview takes approximately 1 hour to administer. PD features were rated positive if they were pervasive, associated with significant functional impairment or subjective distress, did not occur exclusively during or were better explained by Axis I disorders, and were present for at least two years. This is one year longer than what the DSM-IV-TR requires for diagnosing PDs in adolescence, but in accordance with diagnostic practice applied in other studies on PD in adolescents [24,54]. Also, the DSM-IV criterion B (the individual is at least 18 years) for antisocial PD was waived [55].

#### Schedule for affective disorders and schizophrenia for schoolage children-present and lifetime version

The Schedule for Affective Disorders and Schizophrenia for Schoolage Children-Present and Lifetime Version (K-SADS-PL) [56] is a semi-structured diagnostic interview for assessment of current and past psychopathology in children and adolescents, aged 6 to 18 years, according to DSM-IV criteria. In the present study we only assessed for current psychopathology, and did not include family members as informants. Interrater agreement in a randomly chosen subset of the sample (*n* = 20) was excellent, with kappa and ICC values for categorical agreement on specific diagnoses and dimensional agreement on the number of emotional and alcohol and substance use disorders ranging from 0.77 to 0.86.

#### Lifetime history of aggression

The Life History of Aggression: Self-Report (LHA:SR) [57] questionnaire was used to assess aggression tendencies. The LHA:SR is an 11-item self-report instrument measuring lifetime history of actual aggression. The questionnaire consists of three subscales measuring: (1) overt aggression; (2) negative consequences of aggressive behaviours; and (3) self-directed aggression, which includes suicide attempts and self-injurious behaviours, as well as a Total scale score. Items are scored on a 1-to-5 Likert scale. In order, alpha coefficients for the LHA:SR Total, Aggression, Negative Consequences, and Self-Directed Aggression scales, in this study, were 0.87, 0.90, 0.70, and 0.26, indicating acceptable internal consistency. The very low alpha coefficient for the Self Directed Aggression scale was expected because it only consists of two items, and the alpha statistic is to some extend a function of the number of items in a scale.

#### Verbal IQ

The vocabulary subtest of the Wechsler’s scales: Wechsler Intelligence Scale for Children-Third Edition (WISC-III) [58] and Wechsler Adult Intelligence Scale-Third Edition (WAIS-III) [59] were used to assess verbal comprehension and intelligence in participants. Younger participants (aged 16 or younger) were assessed with the WISC, and older participants (aged 17 and above) were assessed with the WAIS. The vocabulary subtest is a test of accumulated verbal learning, generally reflecting the nature and level of a persons schooling and learning environment. The scale is reliable, and is the single best subtest indicator of general intelligence [60]. In this study, alpha coefficients for the vocabulary subscales of the WISC-III and WAIS-III were 0.84 and 0.86 respectively, suggesting adequate internal consistency. Interrater agreement in a subset of the sample (*n* = 20) was excellent (ICC = 0.82).

#### Ratings of ethnicity

To rate ethnicity we followed the guidelines used by Statistics Denmark, which is an institute under the Ministry of Economic and Internal Affairs, publishing national statistics. According to their criteria, a person rated Danish is a person with at least one parent born in Denmark and with a Danish citizenship. Persons rated Immigrant are born outside of Denmark, and both parents are born outside of Denmark or both parents have citizenship in other countries than Denmark. Finally, to be rated Descendant, the person is born in Denmark and both parents are born outside of Denmark or both have citizenship in other countries than Denmark.

### Ethics

This study was approved by the Danish Data Protection Agency and the Research Ethics Committee for Region Zealand acting under the Danish Act on a Biomedical Research Ethics Committee System and the Processing of Biomedical Research Projects. All participants gave informed consent in writing. Neither staff nor participants were offered remuneration for participation.

### Procedure

Eligible participants were enrolled consecutively in the study and randomly allocated to either one of two groups in blocks of five to ensure equal-sized groups. Baseline screening with the SAPAS-AV was performed by staff (8 social workers and 3 psychologists) in Group 1 and a member of the research team (MK) in Group 2. Shortly after the same member of the research team performed all the other research assessments at baseline, and all follow-up screenings with the SAPAS-AV in both groups. Baseline administration of the SAPAS-AV was done first, followed by K-SADS, WISC/WAIS vocabulary subtest, LHA:SR, and SCID-II. This order of presentation was chosen to ensure that knowledge of SCID-II status would not bias baseline SAPAS-AV assessments, and to maximise time length between SAPAS-AV and SCID-II assessment thereby reducing the influence of knowledge of SAPAS-AV ratings on SCID-II ratings in Group 2. At follow-up assessment with the SAPAS-AV, approximately nine days later, participants were instructed not to try and remember or automatically repeat their previous answers, but to respond as felt most appropriate presently. All assessment was done at the sites, and confidentiality was assured for assessments performed by the researcher. For SAPAS-AV assessments administered by staff confidentiality could not be assured, but because the SAPAS-AV is a screening instrument and not a diagnostic test, and at the time of administration not validated, the results of SAPAS-AV screenings by staff were neither kept nor recorded in participants files or used for decision making, and were treated as confidential, not being shared with other staff, social workers, police or family. Staff administering the SAPAS-AV received approximately 10 minutes of instruction in administering the screen.

SCID-II, K-SADS, and WISC/WAIS Vocabulary subtest interviews were audio taped. After data-collection was finished, a second member of the research team (SB) listened to the recordings and rated them blind to the original ratings of the first researcher, in a subsample of 40 participants for the SCID-II, and in a subsample of 20 for the K-SADS and WISC/WAIS. The two subsamples were randomly selected using Statistical Package for the Social Sciences (SPSS) software. Both researchers were clinical psychologist with more than six years of clinical experience, who had been trained by one of the senior authors (ES) on the SCID-II before data-collection began. This senior author was available for consultation to the first researcher during data-collection for discussing diagnostic ratings.

### Data analysis

We used Receiver Operating Characteristic (ROC) analysis to assess the performance of the SAPAS-AV and determine the optimal cut-off score for predicting a dichotomous criterion diagnosis of any PD (present or absent) on the SCID-II. A ROC curve was obtained by plotting pairs of true positives (sensitivity) against false positives (1-specificity) for all possible cut-scores on the SAPAS-AV. The area under the curve (AUC) provides an estimate of the screen’s overall discriminatory accuracy, when all possible cut-scores are taken into account, and can be interpreted as the probability that a randomly chosen person with PD on the SCID-II will score higher on the SAPAS-AV than a randomly chosen person without the disorder. The ROC curve for a good test deviates statistically significantly from the 45-degree diagonal reference line (AUC = 0.5) of the ROC space, where no discrimination exists, and approaches the Ideal Test Point (AUC = 1), located at the top to the left of the upper-left quadrant of the ROC space, where sensitivity and specificity equals 100% respectively, and the false positive rate is 0% [61]. Following Streiner and Cairney [62] we interpret an AUC < 0.7 as low accuracy, from 0.7 to 0.9 as moderate accuracy, and > 0.9 as high accuracy. In practice, however, clinicians use a single cut-score rather than taking all scores into account. Hence, we also calculated sensitivity, specificity, positive and negative predictive values, and overall diagnostic accuracy [63] associated with different cut-scores on the SAPAS-AV. To test whether the results for the SAPAS-AV in the overall sample had been biased by lack of blinded administration of SCID-II in Group 2 or other factors, we constructed independent ROC curves for the two groups and compared their AUC using the method of Hanley and McNeil [64]. The same analysis was done to test for between-sites variability in the performance of the SAPAS-AV in terms of AUC. Here we only compared the two primary sites of investigation, since the number of participants from the two other sites was too low to allow for statistical comparison. Logistic regression analysis was used, with any personality disorder on the SCID-II as outcome variable, to test whether the association with the SAPAS-AV total score would be influenced by the following covariates: age, ethnicity, number of days at the sites before first screening, verbal IQ, emotional disorders (total number of affective and anxiety disorders on the K-SADS), ADHD, and alcohol and substance abuse disorders. We tested for multicollinearity with the Variance Inflation Factor (VIF), and tolerance statistic. A VIF value below 10 [65] and tolerance statistic above 0.1 [66] is considered acceptable.

The internal consistency of the SAPAS-AV was assessed by computing Cronbach’s alpha for the whole scale and for the total score after omitting each item. To estimate test-retest reliability we compared SAPAS-AV scores at baseline and follow-up, calculating Cohen’s kappa for individual items, and the intra class correlation coefficient (ICC) for the total score. Paired *t*-test was carried out to determine differences between means of SAPAS-AV scores at baseline and follow-up. To assess the accuracy of the SCID-II PD criterion diagnoses, we compared diagnostic variability between the two raters in a randomly selected subsample. Kappa values were used to assess categorical ratings (present/absent) both at the overall level, comparing agreement on whether participants has any (1 or more) PD or not, and at the level of specific personality disorders. Since the kappa statistic is dependent on prevalence [67], it was only calculated for those specific PDs that both raters judged to occur in at least five cases each. To assess interrater agreement on dimensional scores for specific PDs the number of diagnostic criteria rated positive (rated ‘3’) on the SCID-II was summed, and ICC values were computed. We employed Fleiss’ [68] guidelines for interpreting kappa and ICC values (poor agreement: < 0.40; fair to good agreement: 0.40 to 0.75; excellent agreement: > 0.75). Convergent validity of the SAPAS-AV with dimensional scores on the SCID-II, and concurrent validity with the LHA:SR was estimated using Spearman’s correlation coefficient, because some of the variables were non-normally distributed. Spearman correlations are reported according to Cohen’s [69] guidelines: low: < 0.30; moderate: 0.30 to 0.50; large: > 0.50. We use 95% confidence intervals (CI) to characterise statistical uncertainty, and in all analyses significance level was set at 0.05, two tailed. Data was analysed using Statistical Package for the Social Sciences (SPSS) for MAC, version 20, except for ROC-analysis, which was done with MedCalc for Windows, version 12.0.

## Results

### Sample characteristics

**Figure 1:**
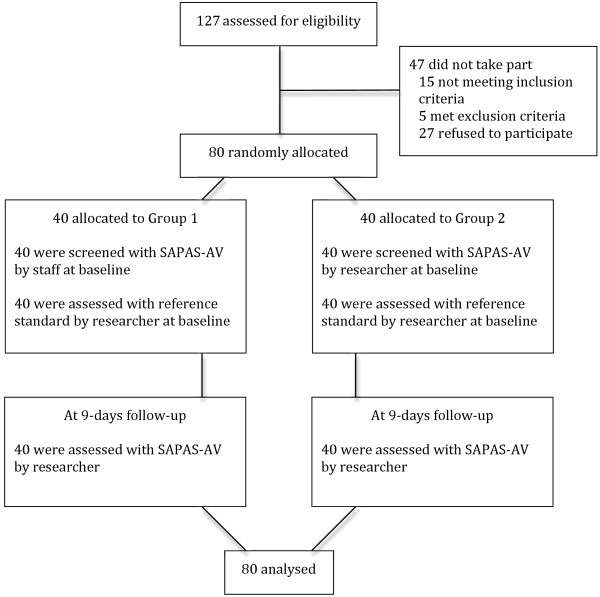
Flow of participants through the study.

From August 2010 to October 2011 127 adolescent boys were assessed for eligibility, and 80 were enrolled in the study. Figure 1 depicts the flow of participants through the study. All participants completed both the screen and reference standard. The time participants had been at the institutions before assessment began ranged from 4 to 549 days, with a median of 12 and mode of 7 days. The mean number of days between baseline administration of the SAPAS-AV and the SCID-II was 0.7 (*SD* = 0.9; range 0 to 4), and 9 (*SD* = 1.1; range 7 to 12) between test-retest with the SAPAS-AV.

The mean age of the sample was 16.5 years (*SD* = 0.8; range 15 to 18). Thirty-nine (49%) participants were either emigrants (*n* = 16) or descendants (*n* = 25), primarily with from the Middle East, Northern Africa, or southern and eastern parts of Europe. Thirty-six (45%) did not have any contact with the education system immediately prior to their placement, and 51 (64%) came from homes where the parents did not live together. The majority of participants (*n* = 67; 84%) were remanded, and the most common offenses were robbery (*n* = 49; 61%) and assault (*n* = 18; 23%). Thirty-five (44%) participants had previously been placed in secure institutions. The most common Axis I disorders were conduct disorder (*n* = 61; 76%), alcohol and substance abuse (*n* = 46; 58%), ADHD (*n* = 18; 23%), anxiety disorders (*n* = 14; 18%), and mood disorders (*n* = 6; 8%). Scores on the WISC/WAIS Vocabulary subtest, measuring verbal comprehension and intelligence, ranged from 6 to 11 with a mean of 8.51 (*SD* = 1.14; Median = 9.00). Sixty-three (79%) participants scored within average range on this test (scores between 8 and 12), and 17 (21%) participants scored below average (≤ 7).

The mean total score was 27.73 (*SD* = 9.33) on LHA, and 2.65 (*SD* = 1.37; range 0 to 6) on the SAPAS-AV at baseline.

The number of PDs in the whole sample and results for interrater agreement on the SCID-II are shown in Table 1. Overall, 52 participants met criteria for at least one PD on the SCID-II, with a mean number of 1.9 specific PDs (*SD* = 0.94; range 1 to 4), yielding a prevalence of 65% for any PD in the sample. The most prevalent specific PDs were Antisocial, Borderline, Narcissistic, and Paranoid. Interrater agreement on the SCID-II in the subsample for a categorical diagnosis of any PD was excellent (kappa = 0.81). At the level of specific PDs, both categorical and dimensional agreement were within good to excellent range.

**Table 1:** Prevalence of personality disorders in the overall sample and interrater agreement on the SCID-II in the subsample

**Personality disorder (PD)**	**Overall sample (*N*** **= 80)**	**Interrater agreement on SCID-II in subsample (*n*** **= 40)**				
	**Number of diagnoses**	**Number of diagnoses**		**Kappa value**	**Dimensional scores**	
		**Rater 1**	**Rater 2**		**ICC**	**95% CI**
Any PD	52	29	30	0.81^***^	_	_
Avoidant	1	0	0	_	0.75^***^	0.58 to 0.86
Dependent	0	0	0	_	0.89^***^	0.80 to 0.94
Obsessive-compulsive	1	0	0	_	0.84^***^	0.72 to 0.91
Paranoid	12	6	5	0.90^***^	0.85^***^	0.74 to 0.92
Schizotypal	3	2	1	_	0.81^***^	0.66 to 0.89
Schizoid	3	1	1	_	0.79^***^	0.63 to 0.88
Histrionic	1	0	1	_	0.71^***^	0.51 to 0.83
Narcissistic	12	9	8	0.78^***^	0.82^***^	0.69 to 0.90
Borderline	17	10	10	0.73^***^	0.90^***^	0.83 to 0.95
Antisocial	47	24	24	0.79^***^	0.87^***^	0.77 to 0.93

Note. PD = Personality Disorder. ^***^p < 0.001.

### Performance of the SAPAS-AV as a screen for any personality disorder on the SCID-II

**Figure 2:**
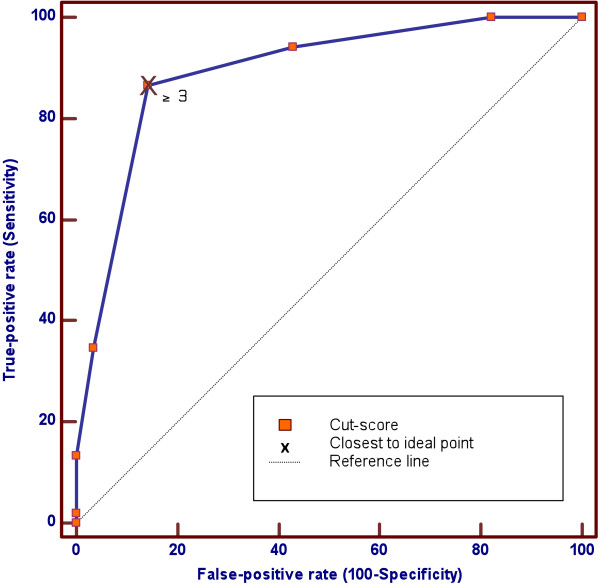
**ROC curve for SAPAS-AV as a screen for any personality disorder in the overall sample.** Note. The ROC curve is significantly different (*p* < 0.0001) from the diagonal reference line, with an AUC of 0.89 (*SE* = 0.04). A cut-score of 3 or more on the SAPAS-AV is closest to the Ideal Test Point, with a sensitivity of 0.87 and specificity of 0.86.

Figure 2 displays the ROC curve for the SAPAS-AV as a screen for a SCID-II criterion diagnosis of any PD in the overall sample. Table 2 shows the results of ROC analysis for the overall sample, both at baseline and follow-up, and for the two groups and two primary sites of recruitment. ROC analysis revealed that the ROC curve, for the overall sample at baseline, was significantly different (*p* < 0.0001) from the non-informative 45-degrees reference line, and had an AUC of 0.89 (*SE* = 0.04; 95% CI = 0.80 to 0.95), indicating moderate overall discriminatory accuracy, when all possible cut-scores are taken into account. Comparison of AUC between baseline and follow-up in the overall sample, and between the two groups and two major recruitment sites respectively, revealed no statistically significant differences. This suggests that the overall discriminatory performance of the SAPAS-AV was moderate and similar from baseline to follow-up, in Group 1 and Group 2, and at the two primary sites.

**Table 2:** ROC analysis of the SAPAS-AV and subgroup comparison of AUC

**Sample**	**AUC (95% CI)**	**Standard error**	**Comparison of AUC difference**
			***p***^**1**^
Whole sample at baseline (*N* = 80)	0.89^****^ (0.80 to 0.95)	0.04	0.74
Whole sample at follow-up (*N* = 80)	0.90^****^ (0.81 to 0.96)	0.03	
Group 1 (*n* = 40)	0.89^****^ (0.75 to 0.97)	0.06	0.93
Group 2 (*n* = 40)	0.89^****^ (0.75 to 0.96)	0.05	
Site 1 (*n* = 34)	0.86^****^ (0.70 to 0.96)	0.07	0.47
Site 2 (*n* = 38)	0.92^****^ (0.78 to 0.98)	0.05	

Note. ^****^AUC is significantly different from the non-informative line (AUC = 0.50) at the level of *p* < 0.0001. ^1^Two-tailed z-test comparing differences between AUC for the two ROC-curves.

The performance of the SAPAS-AV at baseline in the overall sample, for a range of cut-scores, is presented in Table 3. A cut-score of 2 and 3 correctly classified more than 80% of the participants. From a psychometric perspective, a cut-score of 3 is optimal, because it offered the best balance between sensitivity (0.87) and specificity (0.86), correctly classified the highest number of participants (86%), and had the highest agreement with the SCID-II (kappa = 0.71).

**Table 3:** Sensitivity and specificity for different cut-scores on the SAPAS-AV

**Cut-off score**	**Sensitivity (95% CI)**	**Specificity (95% CI)**	**Positive predictive value (95% CI)**	**Negative predictive value (95% CI)**	**Overall Diagnostic Accuracy (%)**	**Kappa value for agreement**
≥ 2	0.94 (0.84 to 0.99)	0.57 (0.37 to 0.76)	0.80 (0.68 to 0.89)	0.84 (0.60 to 0.97)	81	0.56^***^
≥ 3	0.87 (0.74 to 0.94)	0.86 (0.67 to 0.96)	0.92 (0.80 to 0.98)	0.77 (0.59 to 0.90)	86	0.71^***^
≥ 4	0.35 (0.22 to 0.49)	0.96 (0.82 to 1.0)	0.95 (0.74 to 1.0)	0.44 (0.32 to 0.58)	56	0.24^**^
≥ 5	0.14 (0.06 to 0.26)	1.0 (0.88 to 1.0)	1.0 (0.59 to 1.0)	0.38 (0.27 to 0.51)	44	0.10^*^

Note. ^*^p < 0.05. ^**^p < 0.01. ^***^p < 0.001.

### Specificity of the SAPAS-AV as a predictor of PD-status on the SCID-II

Table 4 displays the results of the logistic regression analysis. Multicollinearity in the model was not a problem (VIF ranged from 0.58 to 0.91, and Tolerance from 1.10 to 1.72). The SAPAS-AV remained significantly associated with any PD on the SCID-II (*p* < 0.001) after controlling for age, ethnicity, number of days at the secure sites before first testing, verbal IQ, emotional disorders, ADHD, and alcohol and substance abuse disorders. Alcohol and substance abuse were the only co-variate to be additionally and significantly associated with any PD (*p* = 0.04).

**Table 4:** Logistic regression of the SAPAS-AV on any personality disorder on the SCID-II

**Predictor**	**ß (SE)**	**Wald's*X***^**2**^	***df***	***p***	**Exp(ß)**	**95% CI for Exp(ß)**	
						**Lower**	**Upper**
SAPAS-AV total score	2.55 (0.66)	14.78	1	0.001	12.81	3.49	46.99
Age	0.27 (0.64)	0.18	1	0.67	1.31	0.37	4.64
Ethnicity	0.90 (1.17)	0.59	1	0.44	2.46	0.25	24.23
Time at institution	0.01 (0.01)	1.10	1	0.29	1.01	0.99	1.04
Verbal IQ	−0.21 (0.48)	0.19	1	0.67	0.81	0.32	2.08
Emotional disorders	2.36 (1.30)	3.31	1	0.07	10.57	0.83	134.06
ADHD	0.98 (1.31)	0.56	1	0.45	2.67	0.21	34.84
Alcohol/substance abuse	1.90 (0.92)	4.27	1	0.04	6.65	1.10	40.12

Note. Goodness-of-fit test (Hosmer & Lemeshow) X2= 9.38, df(8), p = 0.31. R2 = 0.55. Nagelkerke R2 = 0.76. Model X2 = 64.00, df(8), p < 0.001.

### Internal consistency and test-retest reliability

The results of the reliability analysis for the overall sample are shown in Table 5. The alpha coefficient for the total scale score was 0.62. Items 6, 7, and 8 were least consistent with the total score.

**Table 5:** Reliability of the SAPAS-AV

**Item**	**Internal consistency (*N*** **= 80)**		**9 days test-retest reliability (*n*** **= 40)**
	**Item-total correlation**	**Alpha if item deleted**	**Kappa**
1. Difficult making and keeping friends	0.48	0.59	0.82^***^
2. Usually a loner	0.35	0.61	0.84^***^
3. Trusts people	0.60	0.56	0.82^***^
4. Loses temper easily	0.63	0.56	0.85^***^
5. Normally impulsive	0.37	0.62	0.74^***^
6. Normally a worrier	0.31	0.63	0.76^***^
7. Usually dependent	0.10	0.63	0.66^***^
8. Perfectionist	0.23	0.64	0.76^***^

Note. Alpha coefficient for the total score was 0.62. ICC for test-retest reliability on the total score was 0.89. ^***^p < 0.001.

Test-retest reliability for the total score was excellent (ICC = 0.89; 95% CI = 0.83 to 0.93; *p* = 0.001). Kappa values for individual items ranged from 0.66 to 0.85, with a mean kappa value of 0.78, indicating excellent test-retest reliability for most items, except for item 5 and 7, for which test-retest reliability was fair to good. The mean total scores, from baseline (*M* = 2.65; *SE* = 0.15) to follow-up (*M* = 2.49; *SE* = 0.15), was highly correlated, but decreased slightly and statistically significantly, *t*(79) = 2.32, *p* = 0.02, *r* = 0.89. As reported above, however, this slight decrease did not affect the overall discriminatory performance of the SAPAS-AV, considering that there were no significant differences between the AUCs at baseline and follow-up (refer back to Table 2).

### Convergent validity of the SAPAS-AV

The results of the correlation between the SAPAS-AV and SCID-II dimensional PD scores, and the LHA:SR are shown in Table 6. The correlation between the SAPAS-AV and Cluster A and B criteria counts were large and significant (*p* < 0.001), whereas the correlation with Cluster C was low but significant (*p* < 0.05). Correlations between the SAPAS-AV and specific PD criteria counts were all statistically significant and ranged from moderate to large, except for narcissistic which were low but significant, and histrionic, avoidant, and dependent which were low and non-significant. The correlation between the SAPAS-AV and total number of any PD criteria was large and significant. Finally, correlations between the SAPAS-AV and LHA:SR total score were large and significant, and ranged from moderate to large for the three subscales.

**Table 6:** Rank-order correlations between the SAPAS-AV and SCID-II personality disorder criteria count and LHA:SR

**Personality disorder criteria count**	**Spearman’s Rho**
Cluster A criteria	0.57^***^
Paranoid	0.58^***^
Schizotypal	0.44^***^
Schizoid	0.39^***^
Cluster B criteria	0.63^***^
Histrionic	0.10
Narcissistic	0.26^*^
Borderline	0.54^***^
Antisocial	0.50^***^
Cluster C	0.27^*^
Avoidant	0.10
Dependent	0.08
Obsessive-compulsive	0.30^**^
Total number of PD criteria	0.70^***^
**LHA:SR**	
LHA:SR Aggression	0.48^***^
LHA:SR Negative consequences	0.60^***^
LHA:SR Self-directed aggression	0.37^**^
LHA:SR Total score	0.59^***^

Note. PD = Personality Disorder. LHA:SR = Life History of Aggression: Self-Report ^*^p < 0.05. ^**^p < 0.01. ^***^p < 0.001.

## Discussion

Results indicate that the SAPAS-AV is a useful screen in secure institutions for male adolescents offenders, where PDs are common. ROC-analysis showed that the SAPAS-AV has moderate discriminatory accuracy, and that a cut-score of 2 or 3 correctly identified the presence of PD in more than 80% of the participants. A cut-score of 3 or more provided the optimal balance between sensitivity and specificity, correctly classifying PD status in 86% of the participants. Comparison of the performance of the SAPAS-AV in terms of AUC, between the two groups and two major recruitment sites, revealed no statistically significant differences in discriminatory accuracy, suggesting that the performance of SAPAS-AV is robust across sites and between different administrators, and that the estimates for the overall sample has not been critically biased by subgroup differences, or lack of blinded SCID-II administrations. Results from the logistic regression analysis also showed that the ability of the SAPAS-AV total score to predict any PD on the SCID-II remained significant, even after controlling for clinically and demographic covariates, which we suspected could influence its discriminatory abilities. The only co-variate to be additionally associated with any PD on the SCID-II was alcohol and substance abuse. We would not interpret this result as suggestive of a confounding effect, since a substantial body of research suggests that PDs commonly co-occur with substance abuse [70].

The internal consistency of the SAPAS-AV was somewhat low (alpha = 0.62), albeit comparable to the original instrument in adult samples [44,47], considering that alpha values are generally required to be at least 0.70 in order for a scale to be considered reliable [71]. However, it should be taken into account that alpha values depend on the number of scale items and the dimensionality of the construct being measured [72]. In light of this, we therefore argue that the internal consistency of the SAPAS-AV is acceptable, given the scale’s limited number of items and the multidimensional nature of PDs [43]. Items 6, 7, and 8 were least consistent with the total scale score, suggesting that eliminating these items could improve the internal consistency of the scale. This improvement of alpha (raising it to 0.63 or 0.64), however, would only be marginal, and probably without any practical value. Also, inspection of the content of these three items suggest that they primarily target features of PDs associated with Cluster C personality disorder criteria, with which the SAPAS-AV only correlated weakly in terms of their respective dimensional scores on the SCID-II, and statistically non-significantly for avoidant and dependent personality disorder criteria. Nevertheless, in adults with substance dependence, the SAPAS has been found to correlate well with Cluster C PD criteria [48]. Hence, we are hesitant to remove any of the Cluster C related items from the scale, until further research has been done on the SAPAS-AV in samples where Cluster C PDs are more prevalent.

Test-retest reliability for the total score on the SAPAS-AV was excellent over a period of nine days. However, this result could be biased by the fact that it was the same member of the research team who performed all SCID-II assessments, and, in Group 2, also the baseline SAPAS-AV assessments, and later performed all follow-up assessments with the SAPAS-AV. Accordingly the follow-up assessments were not undertaken in a blind manner. The mean scores on the SAPAS-AV at baseline and follow-up were strongly correlated, but there was also a slight but statistically significant decrease in mean scores over time. Since comparison of the AUC for the SAPAS-AV at baseline and follow-up showed no significant differences, the overall discriminatory performance was unaffected by this decrease. Future research could address this issue by incorporating a longer time interval between test-retest, and use blinded raters, to test whether the increase might affect the discriminatory ability of the SAPAS-AV for longer periods.

The findings suggest that, as is the case with the SAPAS, the SAPAS-AV is viable as a dimensional measure of PD. Apart from Cluster C PDs, with which it correlated weakly, its association with Clusters A and B, and total number of PD criteria met was large. The large correlation between the SAPAS-AV and LHA:SR, suggest that the SAPAS-AV might also be of use in the assessment of aggression, including self-directed aggression. Research, using a prospective follow-up component, is needed to examine how well the SAPAS-AV predicts future aggressive behavior in young offenders.

### Limitations

Despite the encouraging findings, some methodological limitations should be taken into regard when interpreting the results. The main limitation was lack of blinding in the undertaking of SCID-II assessments. Apart from baseline administration of the SAPAS-AV in Group 1 by staff at the institutions, for practical and economic reasons, all other assessments were done by only one member of the research team, which introduces the risk of test and diagnostic review bias [73]. We tried to assess whether this, or other sources of variability, had biased the results by comparing the performance of the SAPAS-AV at baseline between the two groups and found there was no significant differences in the overall discriminatory abilities of the SAPAS-AV between the groups. However, in conducting this subgroup analysis, we acknowledge that our statistical power was limited by the small sample size. Given that fact that there were no differences in the performance of the SAPAS-AV between the two groups, and since the SAPAS-AV requires no interpretation on behalf of the administrator, and the SCID-II is of a semi-structured nature, we think it unlikely that our overall results have been significantly biased. Furthermore, since our sample only consisted of adolescent boys, with the vast majority being remanded, and with a high prevalence of PDs, it is unknown whether the psychometric properties of the SAPAS-AV found in this study, might be generalised to different settings and populations. It is therefore important that future research aims at validating the SAPAS-AV in other settings and populations, and includes girls. Finally, we emphasise that the present results for the SAPAS-AV as a screen for PD amongst incarcerated adolescent boys, should be considered preliminary until they have been replicated in similar settings and populations.

### Potential applications of the SAPAS-AV

We envisage that the SAPAS-AV could be used for both clinical and research purposes. Clinically, the SAPAS-AV can be used as a brief staff-administered routine screen to identify young people in need of further detailed specialist assessment for PD. Furthermore, the SAPAS-AV can be used in epidemiological research on PD in young people, as first-stage screen in a two-stage procedure for case-identification [74]. In general, we would advise using a cut-score of 3 or more on the SAPAS-AV to identify young people in need of further assessment of PD. Depending on setting and the relative importance of sensitivity and specificity however, users might want to adopt other cut-scores. Hence, in settings with a high prevalence of PD, as might often be the case in juvenile justice settings, users might opt for using a cut-score of 4 or more, to enhance specificity, effectively ruling persons in need of further personality assessment out rather than in. Timing of assessment is of some importance and there are pros and cons associated with delaying assessment. Using the SAPAS-AV as a screen for PDs soon after inception could potential inflate estimates of PD. On the other hand, assessment of PD on inception might have important implications for decisions regarding treatment, risk and needs assessment, and case-management [75].

In developing the SAPAS-AV we hope to facilitate early identification of PD amongst clinicians working with adolescent male offenders. Still, early identification and diagnosis is not an end in itself, but rather a means for evidence based clinical decision-making and case-management, risk and needs assessments, and the development and implementation of early intervention programs for PD in adolescence. Before implementing the SAPAS-AV as part of a routine screening programme, practitioners needs to give due and balanced consideration to potential useful as well as harmful consequences to the young people being screened. A major concern is negative consequences of labelling. Indeed, using the SAPAS-AV as a screen in secure institutions would likely lead to more diagnoses of PD amongst young offenders, with potential for increased stigma. However, we think that the detection of serious personality problems in young offenders is important, as it helps to identify an important risk variable and facilitate better person-centered case management and treatment [76,77]. We think such benefits considerably outweigh any perceived disadvantage of screening. Moreover, it is worth emphasising that none of the young people who participated in this study voiced concerns about the nature or purpose of the screen, suggesting it was acceptable to the young people. Finally, we would like to stress that the SAPAS-AV should not be used as a substitute for rigorous diagnostic assessment of PD, nor should any clinical or judicial decisions be made based solely on the SAPAS-AV score.

## Conclusion

This preliminary study is the first to examine the validity and reliability of the SAPAS-AV as a screen for PD in a sample of incarcerated adolescent boys. The results provide preliminary evidence of the validity, reliability, and clinical utility of the SAPAS-AS as a brief routine screen for PD amongst incarcerated adolescent boys.

## Appendix 1 The Standardised Assessment of Personality–Abbreviated Scale: Adolescent Version (SAPAS-AV)

**Instructions to administrators**: Only circle Y (yes) (or N (no) on item 3) if the respondent confirms the follow-up question that the description applies *most of the time* and *in most situations*.

1. In general, do you have difficulty making and keeping friends?....................Y/N

(yes = 1, no = 0)

2. Would you normally describe yourself as a loner?...............................Y/N

(yes = 1, no = 0)

3. In general, do you trust other people?.........................................Y/N

(yes = 0, no = 1)

4. Do you normally loose your temper easily?...................................Y/N

(yes = 1, no = 0)

5. Are you normally an impulsive sort of person, who generally acts before thinking?.....Y/N

(yes = 1, no = 0)

6. Are you normally a worrier?.......................................................Y/N

(yes = 1, no = 0)

7. In general, and compared to your peers, do you depend on others a lot?..............Y/N

(yes = 1, no = 0)

8. In general, are you a perfectionist?...........................................Y/N

(yes = 1, no = 0)

## Competing interests

The authors declare they have no competing interests.

## Authors' contributions

MK planned and designed the study, collected the data and conducted all assessments (except for baseline SAPAS-AV screenings in Group 1), performed the statistical analysis, and drafted the manuscript. PM supervised the study design and the adaptation of the SAPAS-AV. SB conducted ratings used for assessment of interrater agreement. ES supervised study design and assessments. All authors contributed to the writing and revision of the manuscript. All authors read and approved the final manuscript.

## Pre-publication history

The pre-publication history for this paper can be accessed here:

http://www.biomedcentral.com/1471-244X/12/94/prepub
